# Roles of lipid metabolism in keloid development

**DOI:** 10.1186/1476-511X-12-60

**Published:** 2013-05-01

**Authors:** Chenyu Huang, Rei Ogawa

**Affiliations:** 1 Department of Plastic, Reconstructive and Aesthetic Surgery, Nippon Medical School, 1-1-5 Sendagi Bunkyo-ku, Tokyo, 113-8603, Japan; 2 Department of Plastic Surgery, Meitan General Hospital, Beijing, China

**Keywords:** Lipid metabolism, Pathological scar, Keloid, Inflammation, Mechanotransduction

## Abstract

Keloids are common cutaneous pathological scars that are characterised by the histological accumulation of fibroblasts, collagen fibres, and clinically significant invasive growth. Although increasing lines of research on keloids have revealed genetic and environmental factors that contribute to their formation, the etiology of these scars remains unclear. Several studies have suggested the involvement of lipid metabolism, from a nutritional point of view. However, the role that lipid metabolism plays in the pathogenesis and progression of keloids has not previously been reviewed. The progress that has been made in understanding the roles of the pro- and anti-inflammatory lipid mediators in inflammation, and how they relate to the formation and progression of keloids, is also outlined. In particular, the possible relationships between mechanotransduction and lipid metabolites in keloids are explored. Mechanotransduction is the process by which physical forces are converted into biochemical signals that are then integrated into cellular responses. It is possible that lipid rafts and caveolae provide the location of lipid signaling and interactions between these signaling pathways and mechanotransduction. Moreover, interactions between lipid signaling pathway molecules and mechanotransduction molecules have been observed. A better understanding of the lipid profile changes and the functional roles lipid metabolism plays in keloids will help to identify target molecules for the development of novel interventions that can prevent, reduce, or even reverse pathological scar formation and/or progression.

## Introduction

Keloids are a type of cutaneous pathological scar. Regarding their clinical characteristics, they usually “invade” into the neighboring healthy skin with a leading edge that is often erythematous and pruritic, and they are difficult to treat. In terms of their pathological characteristics, they exhibit the accumulation of fibroblasts and collagen fibres that are hyalinised and eosinophilic [1]; as such, they can be considered as a fibroproliferative disorder of the skin [2]. Increasing research into the etiology of keloids has mainly centered on genetic factors [3] and local factors, such as wound tension [4], sebum secretion [5], and neurogenic inflammation [6]. However, several studies by Louw have suggested, mainly from the nutritional point of view, that essential fatty acids may be involved in the formation and progression of keloids [7-10], which has galvanised research into the contribution of nutrition in keloid development and progression. However, the roles that lipid metabolism may play in keloid pathogenesis have not previously been reviewed.

Lipids mainly constitute the storage fat triglyceride and the lipoids. The lipoids include a wide range of functionally-active molecules, such as phospholipids, glycolipids, and cholesterol. In the skin, they are mainly found in biomembrane structures (e.g., phospholipids) and the stratum corneum (e.g., ceramides), where they serve as membrane constituents and provide secondary messengers. More importantly, they are functionally involved in local inflammation and intracellular signal transduction. A better understanding of the lipid profile changes and functional roles of lipid metabolism in keloids will help to identify target molecules for the development of novel interventions that can prevent, reduce, or even reverse pathological scar formation and/or progression.

### Constitutional changes in lipid profiles in keloid

When comparing the mean concentrations of lipids, the ratio of triglycerides in keloidal skin is 60% of that in normal skin, although it has similar ratio of cholesterol and fatty acids. The cholesterol ester and wax ester levels are reduced to 67% and 20% of the levels in normal skin, respectively [11]. This may reflect changes in the mechanisms that cause these esters to accumulate in the skin, namely their transfer from the dermis, adipose tissue and serum, their synthesis in the epidermis, and their modification by bacterial enzymes [11,12]. In relation to this, patients with keloids have been found to consume higher levels of dietary linoleic acid (LA) and arachidonic acid (AA) than recommended (7–11 g/d), and they also consume α-linolenic acid (ALA), eicosapentaenoic acid (EPA), and docosahexaenoic acid (DHA) at levels below the recommended 1.1–1.5 g/d [9]. Moreover, keloids bear higher levels of AA than the skin of keloid-prone and non-keloid-prone patients [10]. It may result from (1) excessive dietary intake of AA itself and its precursor LA by patients with keloids, (2) the activated phospholipase A2 (PLA_2_) for AA release from the membrane pool, and (3) the infiltration of lymphocytes and cytokine secretion in the keloid edges which deplete inflammatory competitor of EPA [10]. Moreover, topical glucocorticosteroids, which are often used to treat keloids, can inhibit the synthesis in the epidermis and cultured keratinocytes of the three key stratum corneum lipids, namely cholesterol, fatty acids, and ceramides. Such epidermal lipid synthesis inhibition decreases the levels of lipid precursors for lamellar body formation and lamellar bilayer generation [13]. This observation supports the notion that changes in the lipid composition of keloids may be associated with their progression.

### Relationship between lipid metabolism and inflammation in keloids

Keloids are clinically characterized by chronic inflammation of the leading edges during their invasion into the neighboring healthy skin. This is supported by the presence of infiltrating inflammatory cells on histology [2]. It has been suggested that this prolonged, active inflammatory reaction is due to cyclical skin tension, which stimulates mechanotransduction pathways [14]; it may also induce the production of neuropeptides that promote neurogenic inflammation pathways [6]. Recently, it was suggested that another etiological factor of keloids is altered lipid metabolism, particularly the metabolic processes that relate to essential fatty acids. It is possible that these alterations may promote the inflammatory reaction in keloids.

**Figure 1:**
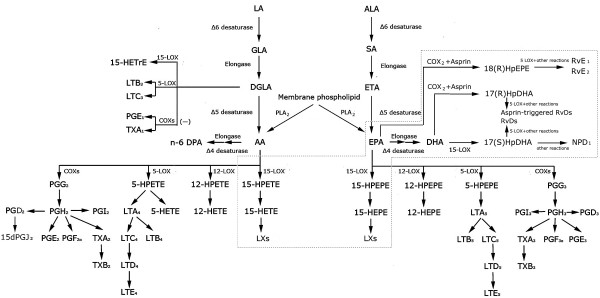
**Overview of the pro- and anti-inflammatory lipid modulators that are generated by lipid metabolism.** Lipid metabolism produces not only the classical pro-inflammatory lipid modulators, namely, the prostaglandins, leukotrienes, and thromboxanes, it also generates anti-inflammatory lipid modulators, namely, lipoxins, protectins, and resolvins (in dashed boxes). The possibility that keloid formation may be promoted by imbalances between the pro- and anti-inflammatory lipid modulators is attracting increasing interest in keloid research. (AA: arachidonic acid; ALA:α-linolenic acid; COX: cycloxygenase; DGLA: dihomo-γ-linolenic acid; DHA: docosahexaenoic acid; ETA: eicosatrienoic acid; EPA: eicosapentaenoic acid; GLA: γ-linolenic acid; HETrE: hydroxyeicosatrienoic acid; HPEPE: hydroperoxyeicosapentaenoic acid; HPETE; hydroperoxyeicosatetraenoic acid; HEPE: hydroxyeicosapentaenoic acid; HETE: hydroxyeicosatetraenoic acid;LA: linoleic acid;LT: leukotriene; LOX: lipoxygenase; LX: lipoxin; NPD_1_: neuroprotectin D1; PG: prostaglandin;PLA_2_: phospholipase A2; SA: stearidonic acid; TX: thromboxane; Rv: resolvin).

High levels of AA are a prominent characteristic of keloids [10]. AA is the source of several downstream products, including the classic eicosanoids: namely, leukotrienes (LTs), such as LTB_4_; prostanoids of prostaglandins (PGs), such as PGE_2_; prostacyclins, such as PGI; and thromboxanes (TXs) (Figure 1). These eicosanoids are generally considered to be pro-inflammatory modulators. However, AA is also the source of the non-classic eicosanoids, called lipoxins (LXs), which serve to downregulate inflammation (Figure 1). Notably, although PGE_2_ is classically thought to be pro-inflammatory, it is increasingly being realized that it can act in an anti-inflammatory fashion as well, as it inhibits the production of the pro-inflammatory cytokines tumor necrosis factor-α (TNF-α) and interleukin-1β (IL-1β) [15] and improves the production of the anti-inflammatory lipoxins by inducing 15-lipoxygenase (15-LOX) [16,17]. This is in accordance with the fact that, compared to normal human dermal fibroblasts, keloid-derived fibroblasts (KFs) have a diminished capacity to produce PGE_2_[18]. Moreover, KFs produce less PGE_2_ in response to macrophage migration inhibitory factor (MIF) and have lower E prostanoid receptor 2 levels [19]. Given that PGE_2_ enhances MMP-1 expression [20], the reduced PGE_2_ levels in keloids may be responsible for the decreased MMP-1 production by keloids and their subsequent accumulation of extracellular matrix (ECM). Another lipid mediator, called cyclopentenone prostaglandin 15-deoxy- Δ^12,14^-prostaglandin J_2_ (15d-PGJ_2_), also appears to have both pro-inflammatory and anti-inflammatory activities. These activities are dependent on its concentration: at low concentrations, it enhances eotaxin-induced chemotaxis in eosinophils; but at high concentrations, it inhibits eosinophil survival by inducing apoptosis [21]. To our knowledge, little is known about the roles played in keloids by other products of AA metabolism, such as LT, PGI, and TX.

Given that AA has pro-inflammatory effects and EPA and its downstream product, DHA, can inhibit the production of inflammatory cytokines, such as IL-6 or TNF-α [22-24], Louw postulated that keloids may be treated by modulating the membrane FA composition, namely to reduce AA levels by supplementing the diet with GLA, DGLA, and EPA; it was proposed that this would restore the levels of AA precursors and inflammatory competitors and reduce the excessive release of AA [10]. This notion was supported by the fact that snake oil, which has high levels of LA, inhibits the growth of human KFs *in vitro*[25]. Moreover, this traditional African medicine, when consumed, both prevents and treats keloids [10].

Given that keloids, as fibroproliferative disorders, are to some extent the product of an imbalance between pro-and anti-inflammatory processes, attention should be paid to the “good” lipid mediators, namely, those that resolve and antagonize inflammation and even show antifibrotic effects, with the help of 5-LOX and 15-LOX. These “good” mediators include the LXs, protectin Ds (PDs), and resolvins (Rvs). Of these, the AA- and EPA-derived LXs and the aspirin-triggered LXs (ATLs) have endogenous anti-inflammatory, proresolving and anti-fibrotic functions [26,27]. Supporting evidence comes from studies on experimental dermal fibrosis: in this model, 15-LOX and its LX metabolites play a prominent anti-fibrotic role [28]. LXA_4_ and ATLa also attenuate experimental renal [29] and pulmonary [27] fibrosis, respectively. Similarly, the Rv and PD lipid mediators possess potent local anti-inflammatory and resolution properties [30]. However, although they can inhibit fibrosis in organs such as the kidney [31], little is known about their influence in skin and keloids. The mechanisms that cause switching between the lipid mediator classes, namely, from the generation of pro-inflammatory PGs and LTs to the production of anti-inflammatory LXs, PDs, and Rvs, also remain to be clarified. This information will help to identify the pathways that can be targeted to inhibit or prevent keloids.

### Relationship between lipid-derived secondary messengers and keloids

Lipids are not only constituents of the skin and can act as pro- and anti- inflammatory factors, they are also the source of secondary messengers that can influence the cellular events that are responsible for the development of keloids. These secondary messengers include diacylglycerol (DAG), ceramide and AA. In terms of keloid development, the most significant of these molecules is DAG. DAG binds to the cysteine-rich domains of protein kinase C (PKC) [32] and high levels of DAG correlate with persistent activation of PKC [33], which has been shown to contribute to the proliferation of fibroblasts in keloids [10]. Another important molecule in keloid development is ceramide, whose structure is similar to that of DAG. Ceramide is a lipid secondary messenger that is derived from cell membrane sphingomyelin and influences apoptosis pathways that are induced by stimuli such as Fas antigen [34,35]. Thus, ceramide mediates extracellular signals. Compared to normal dermal fibroblasts, KFs appear to be resistant to ceramide-induced apoptosis because they over-express insulin-like growth factor I (IGF-I) receptor [36]. It may also be that Fas-mediated signals are not transduced to ceramide in keloids and ceramide is not activated in anti-Fas-stimulated KFs [37]. With regard to AA, although it produces inflammatory factors and has long been known to participate in messenger systems that involve kinases, such as PKC, PKA, and mitogen-activated protein kinase (MAPK) [38], whether it can serve in KFs as secondary messengers remains unclear. Moreover, little seems to be known about the keloid-forming roles of other possible lipid-derived second messengers, such as phosphatidic acid (PA), lysophosphatidic acid (LPA), and inositol-1,4,5-triphosphate (IP_3_).

### Interactions between lipids and mechanotransduction in keloids

Lipids are essential components of the membrane, which is the interface between the extracellular and intracellular compartments. This fact, together with the fact that lipids and their endogenous metabolites provides pro- and anti-inflammatory factors and secondary messengers, strongly supports the notion that lipids may influence the intracellular signaling pathways in keloids. Keloids are fibroproliferative skin disorders that appear increasingly to have a close relationship with local mechanical forces, as indicated by their preferred distribution in high-tension areas, their responsiveness to tension-reduction therapy, and their high levels of mechanotransduction pathway signaling [14,39]. Mechanotransduction is the process by which physical forces are converted into biochemical signals that are then integrated into cellular responses [40,41]. In keloids, the mechanotransduction pathways may involve the transforming growth factor-β (TGF-β)/Smad, MAPK, integrin, RhoA/ROCK, Wnt/β-catenin, and TNF-α/nuclear factor kappa-light-chain-enhancer of activated B cells (NF-κB) pathways [42]. Therefore, research into possible intersections between these mechanotransduction pathways and lipids is likely to identify pathways and potential molecules that could be targeted by anti-keloid therapeutic approaches.

Several lines of research indicated the potential interactions between lipids and mechanotransduction. Lipid rafts are specialized plasma membrane microdomains enriched in cholesterol and sphingolipids, which may serve as signaling compartments [43,44]. As one type of lipid raft, caveolae are flask-shaped plasma membrane invaginations, whose major protein constituent is caveolin-1 [44]. Caveolae have already been proven to be important mechanotransduction sites in osteoblasts [45] and endothelial cells [46]. In endothelial cells, cholesterol depletion prevents the shear-induced activation of MAPKs [46] and caveolin-1 phosphorylation [47], and shear stress causes the phosphorylation of caveolin-1 and its recruitment to integrin sites [48]. Moreover, the β1 integrin-mediated mechanotransduction in endothelial cells is mediated by caveolae domains [47]. In osteoblasts, disruption of cholesterol-rich plasma membrane compartments significantly reduces hydrostatic pressure- and shear-induced mechanotransduction. Moreover, restoration of plasma membrane rafts causes osteoblasts to regain their mechanotranducing properties [45]. The relationship between lipid rafts/caveolae and mechanotransduction in keloids is just being recognized. Evidence for this is that KFs have markedly decreased caveolin-1 expression and that treatment of KFs with caveolin-1 cell-permeable peptide (cav-1p) inhibited the TGF-β1-induced up-regulation of collagen type I, fibronectin, and α-SMA mRNA and protein expression. Moreover, the inhibitory effect of cav-1p on KF fibronectin production could be blocked by inhibiting the ERK1/2, but not the p38 and JNK pathways [49]. Further research is needed to clarify the relationship between lipids and the active mechanotransduction in keloids.

Lipids and their metabolites may also interact and shape the mechanotransduction pathways in keloids. These lipid molecules could be potential targets for pharmaceutical intervention that aims to prevent or treat keloids. There is evidence that the lipid metabolite PGE_2_ can inhibit keloid formation as follows. First, in KFs, the IL-1β-induced up-regulation of COX-2 expression is impaired, resulting in a diminished stimulation of PGE_2_ secretion by IL-1β. Second, the treatment of KFs with PGE_2_ partially reverses the ability of TGF-β1 to up-regulate the production of collagen types I and III. Third, PGE_2_ inhibits KF cell migration and contraction. Thus, PGE_2_ has both anti-fibroplastic and anti-inflammatory effects on KFs [50]. Vitamin D may also be therapeutic for keloids, as shown by the fact that 1,25-dihydroxyvitamin D3 inhibits the TGF-β1-induced secretion by KFs of matrix proteins such as collagen I, fibronectin, and α-SMA [51]. Moreover, there is evidence that the TGF-β and vitamin D signaling pathways may converge on SMAD [52], or at least cross-talk *via* the binding of vitamin D receptor and Smad3 proteins to their cognate DNA recognition elements [53]. The alkylphospholipid analogue hexadecylphosphocholine (HePC) could also be useful as a therapy because it can inhibit the proliferation of KFs; notably, the enhanced reorganization of collagen I in KF that is induced by HePC relates to the up-regulation of the α2-integrin chain [54].

## Conclusion

In summary, in keloids, lipids not only serve as indispensable skin components, they also actively participate in the chronic inflammation processes that drive the development and progression of keloids and are typically manifested at the edge of keloid skin. Supporting this is that the levels of the metabolic products of the AA and EPA cascades are changed in keloids relative to normal skin. It is also likely that there is an imbalance between the pro-inflammatory PGs and LTs and the anti-inflammatory LXs, PDs, and Rvs within these cascades that promotes inflammation. Lipids also serve as reservoirs of secondary messengers such as DAG and AA that contribute to fibroblast proliferation. Finally, there is evidence that mechanotransduction (an important etiological factor in keloid development) occurs in the lipid rafts and caveolae of the plasma membrane. Moreover, there are interactions between lipids and mechanotransduction pathway molecules, such as PGE_2_, Smads, and integrin. Thus, lipid molecules and their metabolic products may be potential pharmaceutical targets in interventions that aim to prevent, reduce, or even reverse keloid formation and/or progression.

## Abbreviations

AA: Arachidonic acid; ALA: α-linolenic acid; ATLs: Aspirin-triggered lipoxins; COX: Cycloxygenase; DAG: Diacylglycerol; DGLA: Dihomo-γ-linolenic acid; DHA: Docosahexaenoic acid; ECM: Extracellular matrix; EPA: Eicosapentaenoic acid; ETA: Eicosatrienoic acid; GLA: γ-linolenic acid; HePC: Hexadecylphosphocholine; HEPE: Hydroxyeicosapentaenoic acid; HETE: Hydroxyeicosatetraenoic acid; HPEPE: Hydroperoxyeicosapentaenoic acid; HETrE: Hydroxyeicosatrienoic acid; HPETE: Hydroperoxyeicosatetraenoic acid; IGF: Insulin-like growth factor; IL: Interleukin; IP3: Inositol-1,4,5-triphosphate; LA: Linoleic acid; LOX: Lipoxygenase; LPA: Lysophosphatidic acid; LT: Leukotriene; LX: Lipoxin; MAPK: Mitogen-activated protein kinase; NFκB: Nuclear factor kappa-light-chain-enhancer of activated B cells; NPD1: Neuroprotectin D1; PA: Phosphatidic acid; PD: Protectin D; PG: Prostaglandin; PIP: PI-4-phosphate; PIP2: Phosphatidylinositol-4,5-diphosphate; PKC: Protein kinase C; PLA2: Phospholipase A2; PUFA: Polyunsaturated fatty acid; Rv: Resolvin; SA: Stearidonic acid; TGF-β: Transforming growth factor-β; TX: Thromboxane.

## Competing interests

The authors declare that they have no competing interests. There are no non-financial competing interests.

## Authors’ contributions

All authors have made substantial contributions to this work. CH designed and drafted the manuscript after a literature research. RO guided the literature research and the drafting of the manuscript, and made the final corrections to the manuscript. Both authors have read and approved the final manuscript.
